# Examining the Sexual Double Standards and Hypocrisy in Partner Suitability Appraisals Within a Norwegian Sample

**DOI:** 10.1177/14747049231165687

**Published:** 2023-03-27

**Authors:** Leif Edward Ottesen Kennair, Andrew G. Thomas, David M. Buss, Mons Bendixen

**Affiliations:** 1 Department of Psychology, Norwegian University of Science and Technology, Trondheim, Norway; 2 School of Psychology, 7759 Swansea University, Swansea, UK; 3 Department of Psychology, University of Texas at Austin, Austin, TX, USA

**Keywords:** sexual double standards, sexual strategies theory, hypocrisy, short-term sex, sex differences, long-term relationships

## Abstract

Sexual double standards are social norms that impose greater social opprobrium on women versus men or that permit one sex greater sexual freedom than the other. This study examined sexual double standards when choosing a mate based on their sexual history. Using a novel approach, participants (N = 923, 64% women) were randomly assigned to make evaluations in long-term or short-term mating contexts and asked how a prospective partner's sexual history would influence their own likelihood of having sex (short-term) or entering a relationship (long-term) with them. They were then asked how the same factors would influence the appraisal they would make of male and female friends in a similar position. We found no evidence of traditional sexual double standards for promiscuous or sexually undesirable behavior. There was some evidence for small sexual double standard for self-stimulation, but this was in the opposite direction to that predicted. There was greater evidence for sexual hypocrisy as sexual history tended to have a greater negative impact on suitor assessments for the self rather than for same-sex friends. Sexual hypocrisy effects were more prominent in women, though the direction of the effects was the same for both sexes. Overall, men were more positive about women's self-stimulation than women were, particularly in short-term contexts. Socially undesirable sexual behavior (unfaithfulness, mate poaching, and jealous/controlling) had a large negative impact on appraisals of a potential suitor across all contexts and for both sexes. Effects of religiosity, disgust, sociosexuality, and question order effects are considered.

## Introduction

Sexual double standards (SDS) are social norms that permit greater sexual freedom for, or impose greater social opprobrium on, one sex over the other. Traditional reasoning and widespread belief in Western cultures suggests that societies restrict and negatively sanction female more than male sexuality (Baumeister & Twenge, 2002), producing a sexual double standard whereby women are evaluated more negatively than men for engaging in equivalent sexual behavior or expressing sexual agency. These sex differences are captured in the words applied to men and women with highly active sex lives. Men are referred to as “stud,” “player,” or “Lady's man,” while women are referred to as “slut,” “slag,” and “whore” for the equivalent behavior (Buss, 2016).

Previous research has revealed that humans possess a distinct mating psychology for both short-term and long-term mating contexts (MCs) which impacts their mating preferences and choices (Buss & Schmitt, 1993; Thomas & Stewart-Williams, 2018). Yet, considering whether SDS varies as a function of MC is understudied. In the current paper, we examine whether the sexual history of prospective male and female partners is evaluated differently when considering committed long-term relationships versus noncommitted short-term sexual encounters.

## Sexual Double Standards at the Societal and Personal Level

SDS can exist as social norms at a *societal* and a *personal* levels. Studies which use the sexual double standard scale (Crawford & Popp, 2003) repeatedly find evidence of SDS at the societal level—people seem to believe that women are evaluated more negatively than men for sexually active behavior. A recent study of 14 countries examined the status consequences of a variety of behaviors (Buss et al., 2020). Although sexual promiscuity had a negative impact on the status of both men and women, acts of “sexual promiscuity” such as “having sex with two people in one night” had a stronger negative impact on the status and reputation of women than on men. However, such effects do not always translate to a *personal* level as there is no association between beliefs in a societal double standard and personal acceptance of such standards (Bordini & Sperb, 2013; Gómez Berrocal, et al., 2019, 2022; Milhausen & Herold, 1999, 2001; Papp et al., 2015; Ramos et al., 2005).

Various methods have been applied for measuring SDS at a personal level. Crawford and Popp (2003) claimed that a within-subjects design, where participants respond to the same items describing male and female targets performing specific sexual behavior would be the “purest” measure of SDS. Most studies that have compared judgments of male versus female targets find that men are more permissive than are women of sexually active targets of either sex (Jonason & Marks, 2009; Sheeran et al., 1996; Sprecher & Hatfield, 1996; Sprecher et al., 2013).

Studies using between-subject designs have offered insights into factors affecting SDS. For instance, Marks and colleagues (Jonason & Marks, 2009; Marks, 2008; Marks & Fraley, 2005, 2006, 2007) examined SDS by systematically varying information in vignettes describing a man or a woman who had performed one of several forms of sexual behavior. Each target was then subject to a number of evaluations. The findings from these studies do not provide consistent evidence that SDS exist, although one finds some support for responses under high cognitive load or when using more indirect measures (Marks & Fraley, 2006), when evaluating targets in collaborative groups (Marks & Fraley, 2007), and for unusual forms of sexual behavior (Jonason & Marks, 2009). However, the latter finding was unsupported in a recent study showing that women were evaluated *more* favorably than were men when initiating these behaviors (Thompson et al., 2018). Finally, experimental designs using photographs of women who display sexual accessibility or not showed that only women inflicted costly punishment if given the opportunity in games. The authors suggested that SDS is a reflection of intrasexual competition (Muggleton et al., 2019).

Only a small number of individual differences have been studied as potential moderators of the standards men and women hold for their own and others’ sexual behavior. Studies from Spain have found effects of education and social dominance orientation (Gómez Berrocal et al., 2019, 2022) and Sheeran et al. (1996) found that religious individuals held a more traditional SDS. Restrictive attitudes to sex and religiosity are intimately linked (Bendixen et al., 2017) and may result in more control of female sexual behavior, especially those that appear outside committed long-term relationships (e.g., multiple partners, threesomes, traditionally considered sinful behaviors).

Sociosexuality is perhaps the most well-studied personality trait shown to influence sexual standards. Sprecher et al. (2013) found that women and men with unrestricted sociosexuality reported far more acceptance for premarital sexual behavior, but they did not examine whether SDS were affected by individual differences in sociosexuality. Stewart-Williams et al. (2017) also found that participants with an unrestricted sociosexuality were more forgiving of a prospective mate with a high number of past sexual partners. Finally, feelings of disgust may influence how one considers one's own and others’ sexual behaviors, and especially short-term, uncommitted sexual behavior (Tybur et al., 2009). Al-Shawaf et al. (2015) found a specific link between short-term mating strategy and sexual disgust. When considering potential mates for self and friends there might be an effect of moral disgust, too, as this is related to violations of social values and norms.

Further, in a study of Scottish teenagers (Sheeran et al., 1996), women who changed sex partners several times during the year were judged as more irresponsible and lacking in self-respect than men, but only if the respondents self-identified as religious. For judgments of attractiveness and popularity, there was no evidence of an SDS. In a cross-cultural study of American, Russian, and Japanese college students, Sprecher and Hatfield (1996) found some evidence of a traditional double standard among male participants, but this was contingent on level of commitment—for engaged targets there was no SDS. In three Spanish speaking cultures, adherence to SDS was most prevalent in Peru and Ecuador compared to Spain, suggesting that this may be explained by different levels of gender equality (Álvarez-Muelas et al., 2022). Among Canadian students, Milhausen and Herold (2001) found that men were more likely to hold an SDS, thinking more badly about women than men for performing similar sexual behaviors. In contrast, women were more likely to hold a *reversed* sexual double standard by thinking more badly about men more than women. Still, most participants held a single standard. In evaluating a friend's potential date, however, a promiscuous sexual history (i.e., 10 previous partners) led to more negative evaluations for men than women—an SDS in the opposite direction.

Another factor which adds a layer of complexity to personal SDS effects is how one applies one's standards to other people when giving advice. Rudman et al. (2013) had psychology students rate how strongly they had advised same-sex or opposite-sex friends and relatives to accept or reject casual sex offers in the past. The results were in line with a traditional SDS, with less restraint put on men than women. Finally, Sprecher et al. (2013) measured level of permissiveness for premarital sexual behavior for oneself and a typical man and woman. Level of permissiveness was also measured separately for casual and committed relationships. In casual relationships men granted moderately more sexual permissiveness to a hypothetical man than to a hypothetical woman, while women granted marginally more. In committed relationships, neither sex held an SDS.

The extant literature suggests that evidence for SDS effects is neither clear nor straightforward and might depend on a number of factors including specific acts, context (e.g., when giving advice), country-level traits (e.g., sexual egalitarianism), and experimental design. With few exceptions (e.g., Sprecher et al., 2013), the majority of these studies overlook the fact that humans possess a separate long-term and short-term mating psychology. Much like the mating goals, dynamics, costs, and benefits, SDS might vary at the personal level depending on whether one desires and considers a committed partner or a casual one. Applying a sexual strategies perspective to SDS effects might bring greater clarity to their form and function.

### Applying Sexual Strategies Theory to Sexual Double Standards

When and where SDS exist can be informed by evolutionary theory (Buss & Schmitt, 1993; Stewart-Williams & Thomas, 2013; Trivers, 1972). From an evolutionary biological perspective, asymmetries in the costs and benefits of reproduction translate into asymmetries in sexual and familial behavior. For example, the costs of sexual intercourse are much lower for men than women because they have lower obligatory levels of parental investment. As a consequence, men have evolved to be more open to short-term casual sex than women, who tend to be more restricted (Schmitt, 2005). These differences also translate into norms aimed at protecting women over men (Stewart-Williams et al., 2021), which manifest themselves as mate evaluations based on their current sexual behavior and on rumors/gossip of their sexual history (Bendixen & Kennair, 2015; Buss & Dedden, 1990; Schmitt, 2002; Wyckoff et al., 2019), daughter guarding (Kennair & Biegler, 2017; Perilloux et al., 2008), sister guarding (Biegler & Kennair, 2016), and controlling of women's reproductive behavior more generally (Apostolou, 2017). Note, though, that the attitudes to the sexual behavior of daughters, partners, and women in close family, should be quite different to attitudes to the sexual behavior of unrelated women—especially for men in short-term settings.

Sex differences in levels of parental investment depend not only on biological differences, but on contextual ones as well. Sexual strategies theory (SST) makes an important distinction between committed long-term relationships and noncommitted short-term sexual encounters and posits that the sexes have evolved distinct mating strategies to cope with the demands of each (Buss, 1998; Buss & Schmitt, 1993, 2017). In long-term relationships, levels of investment are high in both sexes, minimizing sex differences and leading to a similar mating psychology. In casual uncommitted ones, sex differences in investment are maximized and the mating strategies of each sex are more dissimilar. SST allows us to make several predictions about how someone's sexual history might affect their attractiveness and under what circumstances. For example, it would predict that signals of sexual availability (e.g., promiscuity) would be particularly effective (and judged so by others) when used by women in a short-term context because the main constraint for men's reproductive fitness is identifying and mating with sexually available women. There is ample empirical evidence for this across studies and cultures (Bendixen & Kennair, 2015; Fisher et al., 2009; Kennair et al., 2022; Schmitt, 2002; Schmitt & Buss, 1996). Still, a woman's prior promiscuous behavior may alert men who pursue a mate for long-term relationship to the fact that future children might not be sired by himself, resulting in negative appraisals of her as a potential mate for a long-term committed relationship (Buss, 2016). When men apply sexual availability as a tactic, it is not considered especially efficient in either MC because there are few reproductive benefits for women mating with multiple men, and many potential costs (Bendixen & Kennair, 2015; Schmitt & Buss, 1996).

### Applying Sexual Standards to Self Versus Same-Sex Others

The sexual standards one holds for oneself can differ from one's own *behavior* causing cognitive dissonance. Also, the sexual standards one holds for oneself may differ from the standards one holds for other people causing criticism and harsher judgment. Earlier work on the SDS has primarily considered either effects of appraisals of others or appraisals for oneself. There is therefore little anchoring of the appraisals of others in self-appraisals. We believe that self versus other appraisals, which we denote *sexual hypocrisy*, should be subject to study, and may be informed by evolutionary perspectives. Primarily, we expect to find differences in appraisals of sexual history in potential mates, based on SST, for sex by MC, as an active sexual history is not necessarily negative information about a person. For example, the traditional SDS suggests that men will receive status based on high levels of short-term sexual conquests. While, on the other hand, from an SST perspective, female sexual availability and interest will be assessed more positively by men in a short-term context. However, intrasexual competition is likely to affect how people evaluate same-sex others’ sexual behavior relative to one's own because the cost–benefit analysis associated with a mating decision are often different for the actor than the observer. Thus, we might expect more negative appraisals when considering own potential partner relative to considering a partner for same-sex friend in cases where costs out-weigh the benefits and opposite pattern for sexual behavior where benefits out-weigh the costs. Some indirect empirical supports this form of hypocrisy (Sprecher et al., 2013; Sprecher & Hatfield, 1996). Despite not subject to direct testing, the above findings suggest people display more permissiveness having sex at early stages of a relationship for oneself relative to same-sex others. We expect that men will be more lenient toward some forms of sexual history, and they should be so also for themselves, according to SST. However, this area lacks empirical investigation and theoretical investigation. Nevertheless, adding this aspect, will provide a possibility to consider a specific type of double standard, self versus same-sex others.

We do not currently have any specific hypotheses on how this effect will look though. Primarily we aim to establish this specific appraisal for self. While one might believe that for both sexes reducing other's sexual opportunities in competition with oneself might take priority, especially by men in a short-term setting, our current methodology does not make such a competitive approach clear to participants. Also, there might even be a lack of sexual hypocrisy, no differences in how one appraises partners for self versus same-sex other for the least negative sexual histories, and there might be more risk willingness or sexual liberal attitudes on behalf of others. We will therefore explore this self versus same-sex other constellation.

### Aims and Predictions

In a novel approach to the investigation of SDS, informed by SST, we consider how a prospective partner's sexual history is evaluated in either long-term or short-term MCs using in a large sample of undergraduate students from one of the world's most gender egalitarian, secular, and sexually liberal nations (Bendixen et al., 2017; Grøntvedt & Kennair, 2013). Unlike previous work, we looked at sexual history more broadly by encompassing a diverse range of sexual behaviors, including previous numbers of sexual partners, use of pornography, masturbation, and cheating/controlling/mate poaching behaviors.

We asked participants to judge the suitability of a prospective partner not only for themselves, but for a same and opposite sex friend as well. If men and women are evaluated differently for identical sexual behaviors, this would be indicative of either a traditional double standard (favoring men) or a reversed double standard (favoring women). Differences between evaluations on behalf of oneself and same-sex friend would suggest some level of sexual hypocrisy.

Regarding the sexual history of a potential partner for a friend, we do not expect, based on SST, to find evidence of the traditional SDS for signals of sexual availability (Bendixen & Kennair, 2015; Kennair et al., 2022; Schmitt & Buss, 1996). Instead, we expect a reversed sexual double standard that generalizes to the other domains of sexual behavioral history. We also expect to find that women will react more negatively to a prospective partner's sexual behavioral history than men will (Jonason & Marks, 2009; Rudman et al., 2013).

*Prediction 1*: Regarding the sexual history of a potential partner for one's male or female friend, we do not expect to find evidence of the traditional SDS for direct signals of sexual availability (Bendixen & Kennair, 2015; Schmitt & Buss, 1996). From an SST perspective, we expect a reversed SDS, with women in a short-term context being evaluated less negatively than women in a long-term context and less negatively than men in both MCs. We expect this pattern of evaluations to generalize to the other domains of sexual behavioral history so that the sexual history of women will be evaluated less harshly in a short-term context than in a long-term one and less harshly than men in both MCs.

*Prediction 2:* Compared to men, women will judge a prospective mate's sexual history more harshly and this gender difference will be present when making evaluations for oneself and for same-sex friends (Jonason & Marks, 2009; Rudman et al., 2013).

Religiosity and disgust should be associated with more negative appraisals (Al-Shawaf et al., 2018; Bendixen et al., 2017; Kennair et al., 2018) and reduced likelihood of pursuing a potential partner for oneself. This should also be reflected in the appraisals for same-sex friends. We expect the opposite pattern for sociosexuality as previous research suggests that sociosexuality will be associated with more sexual permissiveness (Sprecher et al., 2013).

*Prediction 3:* Those high in religiosity and disgust, and those with a restricted sociosexuality, will judge sexual history more harshly.

Research question: Our novel approach allows for comparisons of sexual standards one holds for oneself versus same-sex others (i.e., hypocrisy) and how this is affected by participant sex and MC. There is some indirect evidence in prior studies that people report greater permissiveness for self versus others for sex at an earlier stage of a relationship (Sprecher et al., 2013; Sprecher & Hatfield, 1996). We will examine this hypocrisy double standard for sexual permissiveness in this study.

## Methods

### Participants

A convenience sample of Norwegian students (*N* = 1,036) of the Natural, Social, and Human sciences responded to a paper-and-pencil study on Judgement of Partner Attraction in March 2017. To increase homogeneity of the sample, only heterosexual participants (94.6% of the total sample) and students aged 30 years and younger than were included in the data analyses. Heterosexual orientation was determined by participant sex and sexual preference for the opposite sex partners. Following screening procedures, we also removed monotonous (response set) and extreme responses (*n* = 36). The final sample eligible for analyses (*N* = 923) covered women (*n* = 587, age: *M* = 21.9, *SD* = 1.7) and men (*n* = 336, age: *M* = 22.4, *SD* = 1.7) aged between 19 and 30 years.^1^ The majority of the participants reported “single” as their relationship status (women 55%, men 60%).

### Measures

*Target sexual behaviors.* The following procedure was applied for generating items on sexual behaviors: First, we consulted the work of Buss (2013) who outlined relevant sexual behaviors in opposite-sex targets subject to sexual evaluation. These included virginity, lack of sexual experience, having multiple sexual partners, sexual reputation, sexual infidelity, and having an unfaithful mate. Next, we had 10 groups of Bachelor students working in pairs generating additional sexual behaviors through act nomination procedures. After deleting duplicates, each group categorized the behaviors. Through group consensus discussions and under guidance from the project managers a list of 12 distinct sexual behaviors was selected.

When responding to the questionnaire, each participant considered how much each of the 12 sexual behaviors in a male or a female target would affect their appraisals if a *friend of theirs* met up with such a potential partner. Each participant made appraisals for both a male and a female friend. They we also given either a short-term or long-term context in which to answer the questions. For the former, participants had to consider to what extent their friend should pursue a hook up given their initial sexual interest. For the latter, they had to decide to what extent their friend should pursue a long-term relationship. The participants rated their response on a 7-point Likert scale with anchors and mid-point; −3 (*s/he should absolutely not have a one-night stand/get involved in a long-term relationship*), 0 (*inconsequential*), +3 (*s/he should absolutely have a one-night stand/get involved in a long-term relationship*).

Next, they reported on their *own likelihood* of having sex or entering a relationship with such a potential partner and rated their response for each of the 12 items on a 7-point Likert scale with anchors and mid-point; −3 (*it would absolutely reduce the likelihood*), 0 (*inconsequential*), and +3 (*it would absolutely increase the likelihood*). To reduce response set tendencies the order of the 12 behavioral items were scrambled across the three appraisals. To prevent “purer than thou” effects, participants were always asked about friends first. This secures more liberal and less moralistic judgments for oneself (Engeler & Raghubir, 2018).

*Religiosity.* We posed two questions on religiosity. The first reflected personal conviction (“I consider myself religious”) and the second devotion (“I believe it's important to live by religious rules and ideas”). Participants rated their responses from 1 (*strongly disagree*) to 5 (*strongly agree*). The items were strongly correlated (*r* = .61) and scores were multiplied to form a composite measure of religiosity (Bendixen et al., 2017). Higher scores reflect stronger religiosity.

*Disgust.* We applied a 15-item slightly shortened Norwegian version of Tybur et al.’s. (2009) disgust scale. For each of the 15 items the participants rated their responses on a 7-point Likert scale from 0 (*not at all*) to 6 (*extremely*). Internal consistency for the 15-items scale was acceptable (*α* = .78). Scores were summed and averaged. Higher scores indicate more disgust with 0 denoting absolutely no disgust.

*Sociosexuality.* For measuring individual differences in preference for short-term sexual relationships we applied the revised 9-item sociosexual orientation inventory (SOI-R; Penke & Asendorpf, 2008). The internal consistency of the scale was good (*α* = .84). Scoring and scaling closely followed Penke and Asendorpf's (2008) recommendations. Higher scores reflect less restricted sociosexuality (i.e., a stronger inclination for short-term sexual relationships).

### Design

This was a quasi-experimental design where we applied four versions of a questionnaire. The participants were randomly assigned to respond to questions referring to either long-term or short-term MCs, and to one of the two question order versions. In the latter, participants answered about a female friend meeting up with a man followed by a male friend meeting up with a woman or vice versa.

### Procedure

The participants received information about the study orally in classes during a break (and in writing on the first page of the questionnaire). The questionnaire was then handed out to volunteers and returned in a box within 15 min. The students did not receive any course credit or compensation for their participation. To ensure the respondents’ anonymity no personal information was provided. As long as anonymity is secured and the research is not carried out to examine health issues, this kind of research is not subject to ethical approval in Norway. Still, the research was carried out in line with the APA ethical standards.

## Results

### Sexual Acts

Principal component analysis (maximum likelihood) suggested three common factors among the sexual history items. Items measuring prior history of STI, being bisexual, or having been cheated on in a prior relationship had low communalities and was not included in the analyses. Internal consistency for the three scales was acceptable: *promiscuity* (3 items: sex on first date several times, five or more sexual partners last year, had threesome, α_self_ = .76, α_female__friend_ = .70, α_male__friend_ = .72), *self-stimulating* (3 items: frequent masturbating, frequent porn use, regular use of sex toys, α _self_ = .81, α_female__friend_ = .58, α_male__friend_ = .77), and *cheating & controlling* (3 items: been sexually unfaithful, involved in mate poaching, jealous and controlling, α _self_ = .65, α_female__friend_ = .66, α_male__friend_ = .65). The Means and SDs for the outcome variables across sex and mating context are presented in Table 1.

**Table 1.** Means (SDs) for the Nine Outcome Variables Across Mating Context and Participant Sex.

Outcome variable	Short term		Long term	
	Women	Men	Women	Men
Self-suitor				
Promiscuity	−0.58 (0.82)	−0.10 (0.98)	−0.65 (0.72)	−0.69 (0.89)
Self-stimulating	−0.25 (0.64)	0.32 (0.81)	−0.33 (0.64)	0.05 (0.75)
Cheating and controlling	−1.68 (0.79)	−1.28 (0.89)	−2.10 (0.60)	−1.91 (0.66)
Woman target				
Promiscuity	−0.11 (0.73)	−0.02 (0.82)	−0.35 (0.64)	−0.45 (0.77)
Self-stimulating	0.16 (0.67)	0.43 (0.74)	−0.03 (0.51)	0.09 (0.71)
Cheating and controlling	−1.24 (0.80)	−1.11 (0.92)	−1.82 (0.65)	−1.79 (0.66)
Man target				
Promiscuity	−0.21 (0.72)	−0.14 (0.68)	−0.38 (0.68)	−0.40 (0.71)
Self-stimulating	−0.11 (0.58)	−0.02 (0.67)	−0.19 (0.51)	−0.15 (0.61)
Cheating and controlling	−1.33 (0.84)	−1.19 (0.86)	−1.85 (0.69)	−1.76 (0.70)

### Sexual Double Standard

**Figure 1.**
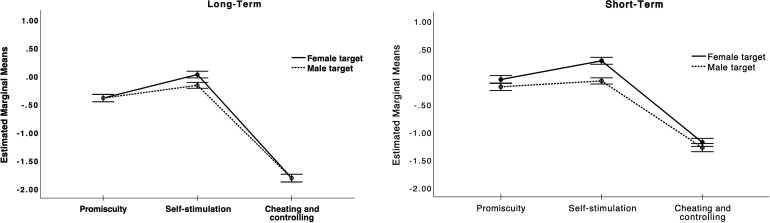
Likelihood of recommending to a friend that they have sex with (right panel) or enter into a relationship (left panel) with a male or female target depending on whether they have a sexual history of promiscuity, self-stimulation, or cheating & controlling.

All within-subject and between-subject effects are presented in Table 2. For testing the sexual double standard (prediction 1), we run a 2 × 3 × 2 × 2 mixed model analysis of variance (ANOVA) (profile analysis) with appraisal (male target vs. female target) and target behavior (promiscuity vs. self-stimulating vs. cheating & controlling) as within-subject factors and participant sex (men vs. women) and MC (short term vs. long term) as between-subject factors. Overall appraisals (profiles) differed strongly for female targets (i.e., a suitor for a male friend) versus male targets (i.e., a suitor for a female friend), such that male targets were less favorably received than female ones, *F*(1, 910) = 155.24, *p* < .001, η*_p_*^2^ = .146. Further, the three types of target behavior received markedly different appraisals, *F*(2, 910) = 2172.95, *p* < .001, η*_p_*^2^ = .705. As we can see from Figure 1, cheating & controlling targets of both sexes were evaluated strongly negative, while the appraisals of promiscuous and self-stimulating targets were closer to neutral.

**Table 2.** Repeated Measures Analysis of the Sexual Double Standard With Appraisal (Male Friend vs. Female Friend) and Behavior (Promiscuous vs. Self-Stimulation vs. Cheating & Controlling) as Within-Subject Factors, and Participant Sex and Mating Context as Between-Subject Factors.

Within-subjects effects	*MS*	*df*	*F*	*p*	η*_p_*^2^
Appraisal	21.45	1	155.24	***	.146
Behavior	1134.14	2	2172.95	***	.705
Appraisal × behavior	6.78	2	73.27	***	.075
Appraisal × sex	0.20	1	1.48	ns	.002
Appraisal × MC	5.44	1	39.38	***	.041
Appraisal × sex × MC	0.43	1	3.11	.078	.003
Behavior × sex	1.13	2	2.17	ns	.002
Behavior × MC	18.88	2	36.17	***	.038
Behavior × sex × MC	0.30	2	0.29	ns	.000
Appraisal × behavior × sex	0.67	2	7.24	.001	.008
Appraisal × behavior × MC	0.17	2	1.79	ns	.002
Appraisal × behavior × Sex × MC	0.02	2	0.24	ns	.000
Error (appraisal)	0.14	910			
Error (behavior)	0.52	1820			
Between-subjects effects appraisal					
Sex	7.18	1	4.98	.026	.005
Mating context	147.54	1	102.31	***	.101
Sex × mating context	3.24	1	2.25	ns	.002
Error	1.44	910			

*Note.* ns =  Not significant, ***p < .001, MS = mean square, MC = mating context (short term vs. long term).

Appraisal profiles differed both across types of behavior and across MC (e.g., significant appraisal × behavior and appraisal × MC interactions). In particular, female targets who were self-stimulating were clearly more positively appraised than their male equivalents, while the gender appraisal differences for promiscuity, and cheating & controlling, were negligible (see Figure 1). However, women with these characteristics were appraised more positively than men in the short-term context but not in the long-term context. Prediction 2 was supported as the between-subjects analyses suggest that women (marginal means: *MM* = –0.63) gave significantly more negative appraisals than men (*MM* = –0.55), and that appraisals in the long-term MC (*MM* = –0.76) were more negative than in the short-term MC (*MM* = –0.42).

Finally, for testing prediction 3 we added religiosity, disgust, and sociosexuality as covariates to examine whether these variables affected appraisals either in the own right or as moderators using standardized scores (Delaney & Maxwell, 1981). Simple sex differences in these covariates were evident for disgust (*d* = –1.04, women higher) and sociosexuality (*d* = 0.47, men higher). There was no sex difference in level of religiosity (*d* = 0.02).

We found significant main effects for all covariates (see Appendix A). Sociosexuality had the strongest effect. The correlations between these covariates and the appraisals split by sexual history type and target sex are shown in Table 3. The correlations showed moderate and positive associations with the outcomes for sociosexuality (*r* = .17 to .35), and negative associations for religiosity (*r* = –.26 to –.10) and for disgust (*r* *=* –.20 to –.15). Participants who were relatively unrestricted in their sociosexuality appraised promiscuity, self-stimulation, and cheating & controlling in target men and women more positively. Participants who scored higher on religiosity and disgust appraised these target characteristics more negatively.

**Table 3.** Zero-Order Correlations Between Covariates Religiosity, Disgust, and Sociosexuality, and Target and Self-Appraisals for Measuring the Sexual Double Standard (n = 897–917).

Target characteristics	Religiosity	Disgust	Sociosexuality
Promiscuous man	–.26	–.17	.35
Promiscuous woman	–.25	–.15	.34
Self-stimulating man	–.18	–.18	.17
Self-stimulating woman	–.17	–.20	.29
Cheating & controlling man	–.10	–.19	.21
Cheating & controlling woman	–.13	–.17	.23

Further, sociosexuality significantly moderated the appraisal of men targets versus women targets profiles, *F*(1, 883) = 12.21, *p* < .001, and the appraisal across the three behaviors profiles, *F*(2, 1766) = 9.64, *p* < .001. As can be seen from Table 3, perceptions of female and promiscuous targets were relatively more influenced by individual differences in sociosexuality than perceptions of male targets, of self-stimulation, and of cheating & controlling behavior.

*Additional analyses of promiscuous behavior.* To provide a more specific test of the SDS (prediction 1), we re-run the above profile analysis for promiscuous behavior with appraisal (male target vs. female target) as within-subject factors and participant sex (men vs. women) and MC(short term vs. long term) as between-subject factors. Overall, a female target's promiscuous behavior was appraised less negatively than similar behavior by a male, *F*(1, 910) = 18.56, *p* < .001, η*_p_*^2^ = .020. This effect was qualified by a significant target sex × MC interaction (*F*(1, 910) = 18.34, *p* < .001, η*_p_*^2^ = .020) suggesting that appraisals for promiscuous behavior was neutral when considering a woman in short-term MC (*MM* = –0.05), negative for a man in the same context (*MM* = –0.18), and equally and markedly more negative for women and men in long-term MCs (*MM* = –0.39). Men and women participants did not differ in their appraisals of promiscuous target behavior.

### Sexual Hypocrisy

To study hypocrisy, we performed an equivalent mixed-model ANOVA as the above, comparing appraisals for oneself (self-suitor) with those of a same-sex friend. This ensures that the sex of the target of the appraisal remains the same (i.e., a male target for women and a female target for men).

**Figure 2.**
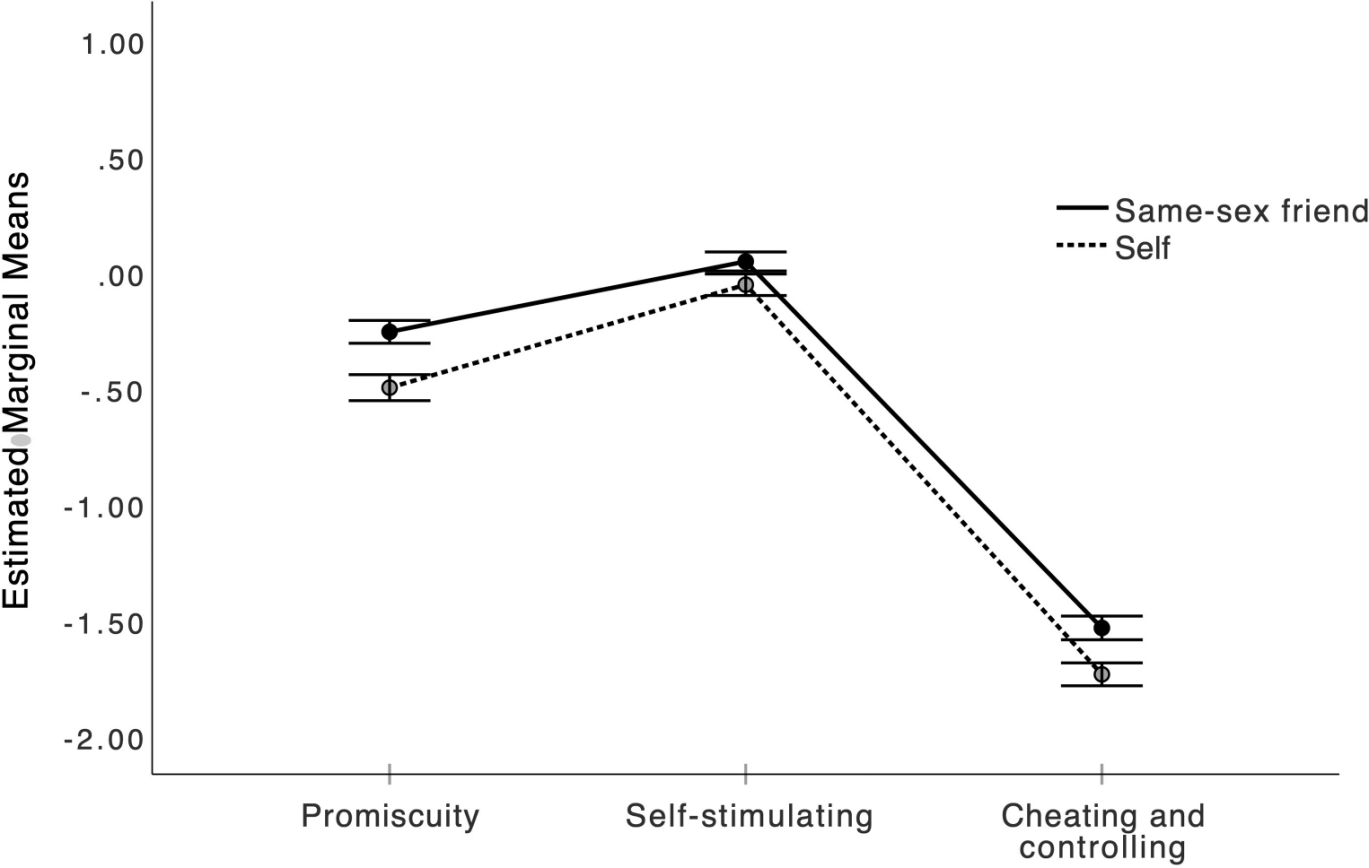
Appraisals of same-sex friend (solid line) and self-reported likelihood (dotted line) of entering into a relationship with a suitor depending on whether they have a sexual history of promiscuity, self-stimulation, or cheating & controlling.

As evident from Table 4, participants made markedly different appraisals for a prospective mate depending on if they were thinking about themselves (self-suitor) or a same-sex friend (friend-suitor). Self-suitor appraisals were clearly more negative relative to friend-suitor ones. These appraisals (profiles) were moderated by participant sex, with women making significantly more differentiated appraisal for self-suitor versus friend-suitor across all types of behavior. Relative to men, women rated the behavior of a prospective mate more negatively for self than for same-sex friend. Appraisals also differed significantly across the three types of behavior (see Figure 2), suggesting smaller appraisal differences for self-stimulating behavior relative to promiscuity, and cheating & controlling behaviors. However, these appraisals were similar across MCs suggesting that self-suitor appraisals were similarly more negative relative to friend-suitor for short-term and long-term mating. The between-subjects analyses suggest that women overall (*MM* = –0.81) gave significantly more negative appraisals (for self and friend) than men (*MM* = –0.54), and that appraisals in the long-term MC (*MM* = –0.85) were more negative than in the short-term MC (*MM* = –0.50). Still, the sex by MC interaction effect suggest that overall short-term appraisals differed significantly more between men and women (*MM* = –0.30 vs. *MM* = –0.70) than long-term appraisals differed between the sexes (*MM* = –0.78 vs. *MM* = –0.92).

**Table 4.** Repeated Measures Analysis of Hypocrisy With Appraisal (Self vs. Same-Sex Friend) and Behavior (Promiscuous vs. Self-Stimulation vs. Cheating & Controlling) as Within-Subject Factors, and Participant Sex and Mating Context as Between-Subject Factors.

Within-subjects effects	*MS*	*df*	*F*	*p*	η*_p_*^2^
Appraisal	40.67	1	233.07	***	.205
Behavior	1215.82	2	2063.28	***	.695
Appraisal × behavior	2.18	2	19.58	***	.021
Appraisal × sex	4.30	1	24.27	***	.027
Appraisal × MC	0.17	1	0.72	ns	.001
Appraisal × sex × MC	0.14	1	0.82	ns	.001
Behavior × sex	8.90	2	15.10	***	.016
Behavior × MC	14.24	2	24.17	***	.026
Behavior × sex × MC	2.63	2	2.23	ns	.002
Appraisal × behavior × sex	0.25	2	2.26	ns	.002
Appraisal × behavior × MC	0.20	2	1.82	ns	.002
Appraisal × behavior × sex × MC	0.61	2	5.49	.004	.006
Error (appraisal)	0.17	904			
Error (behavior)	0.11	1808			
Between-subjects effects appraisal					
Sex	89.78	1	57.84	***	.060
Mating context	148.67	1	95.78	***	.096
Sex × mating context	20.76	1	13.38	***	.015
Error	1.55	904			

*Note.* ns = Not significant, ***p < .001, MS = mean square, MC = mating context (short term vs. long term).

Of the three covariates, only sociosexuality moderated degree of hypocrisy, *F*(1, 880) = 13.29, *p* < .001. The zero-order correlations (Table 5) suggest that sociosexuality was somewhat stronger associated with self-suitor appraisals (*r* = .41) than with friend-suitor appraisals (r = .34), *z* = 1.82, *p* < .07 for promiscuous behavior. Similar to the SDS analysis, sociosexuality had the strongest overall between-subject effect on appraisals of the four covariates, *F*(1, 880) = 105.08, *p* < .001, η*_p_*^2^ =  .107 (see Appendix B).

**Table 5.** Zero-Order Correlations Between Covariates Religiosity, Disgust, and Sociosexuality, and Target and Self-Appraisals for Measuring Sexual Hypocrisy (n = 894–917).

Target characteristics	Religiosity	Disgust	Sociosexuality
Promiscuous (self)	–.29	–.20	.41
Promiscuous (friend)	–.25	–.16	.34
Self-stimulating (self)	–.20	–.31	.32
Self-stimulating (friend)	–.18	–.28	.29
Cheating & controlling (self)	–.11	–.20	.28
Cheating & controlling (friend)	–.13	–.19	.23

## Discussion

The study of SDS has yielded several important results. First, we found a lack of evidence for SDS effects in the traditional direction. Second, we found that people were more discerning of a prospective mate's sexual history in long-term versus short-term contexts and that women were more discerning than men. Third, we found that participants showed some level of hypocrisy—being more cautious when making appraisals for themselves compared to a same-sex friend. Fourth, we found that sexual histories could be reduced to three factors: self-stimulation, promiscuity, and cheating & controlling, and that these factors affected appraisals and were the subjects of SDS and hypocrisy effects in different ways. Finally, we found little evidence that covariates affected the pattern of the results in a meaningful way. We now discuss these key findings in turn.

### A Lack of SDS at the Personal Level

Generally, when people are asked what norms, they believe exist in society, they tend to confirm traditional SDS (the *societal* level). However, when people are asked what attitudes they themselves hold (appraisals at the *personal* level), the pattern can disappear (Crawford & Popp, 2003). Overall, and in line with our predictions, we found a lack of evidence for traditional SDS, and we actually found a reversed sexual double standard in the case of self-stimulation and promiscuous behavior. Rather than women being judged harshly for engaging in porn use, masturbation, and sex toy ownership, they were actually judged to be a slightly more suitable partner for a male friend in short-term contexts, regardless of participant gender, while this aspect of their history had little influence on their suitability as a long-term one. Men in contrast were judged as negatively on the basis of their self-stimulating behavior—more so by women than men and particularly in long-term contexts. Notably, promiscuous women were not evaluated more negatively than promiscuous men in long-term MCs. This pattern was found regardless of perspective (first or third person) and largely generalized to self-stimulating targets and targets with cheating & controlling behavior (unfaithful, jealous, or mate poaching).

### Mating Context and Participant Sex Moderate Appraisals of Sexual History

In this study, we were able to address the fact that little research has considered the role of short-term versus long-term contexts when studying SDS, taking for granted differences in sexual mating psychology that varies both by sex and mating strategy (Buss & Schmitt, 1993). We found that context matters—people rated potential suitors with a sexual history of promiscuity, self-stimulation, and cheating or controlling more harshly if they were considering them as a long-term mate than a short-term one. This difference likely comes from the fact that one of the adaptive problems of those following a short-term mating strategy is identifying opportunities for casual sex. Promiscuity and self-stimulation may act as cues for access and so are tolerated more than in long-term contexts where immediate sexual access becomes less important. Cheating & controlling may have been considered less relevant within short-term contexts for the same reason that kindness is seen as less important in short-term contexts (Li & Kenrick, 2006). Short-term relationships by their very definition make these attributes less relevant—cheating & controlling dynamics tend to happen within ongoing relationships rather than one-night stands.

Another moderator was the sex of the participant. In line with our second prediction, facts about a prospective partner's sexual history generally led to women toward more negative appraisals of than men, regardless of whether they were making judgments for themselves or for same-sex friends. This sex difference was particularly evident for self-stimulating behavior. These sex differences likely reflect the historical asymmetries in the risks associated with sex for men and women. In terms of their reproductive health, having somatic resources “tided up,” and social reputation, the risks of poor sexual decisions for men have historically been much lower than those for women, causing them to evolve to be more cautious about how, when, and with whom they procreate (Buss & Schmitt, 1993).

### Is Sexual Hypocrisy a Specific Form of Sexual Double Standard?

By asking participants to make appraisals for themselves, we were in the unique position to examine sexual hypocrisy. Generally, we found that the participants were less willing to pursue an opposite-sex target following sexual history information but were less cautious when appraising same-sex friends in the same situation. This was also true for men in the short-term context, although these men made relatively fewer negative appraisals for self versus male friend compared to women in both MCs and men in a long-term MC. The reason for this difference we suspect lies with the relative risk to the participant associated with the choice. It would pay to be particularly cautious when making decisions for oneself because one must bear the consequences of that decision. The consequences for even the most beloved friend will always have less of an effect on the self. If this explanation holds then further research should find that appraisals of others’ behavior and choices should track the extent to which negative consequences would impact the decision maker—such as degree of genetic relatedness and interdependence (Apostolou, 2017; Biegler & Kennair, 2016; Perilloux et al., 2008). The traditional double standard is mainly expected to be present in assessment of daughters’, sisters’, mothers’, and wives’ behaviors, not the behavior of sexually available women one is not related to.

Further, appraisals differed for the three behaviors, suggesting that SDS and sexual hypocrisy was not similar for promiscuity, self-stimulation, and cheating & controlling behaviors. The (reversed) SDS effect was more evident for self-stimulation, and more evident in the short-term context, and the sexual hypocrisy effect was stronger for women than for men albeit less pronounced for self-stimulation. Evidently, sexual history is not necessarily best conceptualized as negative information, sometimes sexual history is clearly negative (cheating & controlling behavior), however, self-stimulation is generally not considered negative behavior. The SST perspective highlights the importance of how both sex of actor and MC will influence appraisals of sexual history, for example a woman's sexual availability cues will be assessed more positive for men in a short-term setting than men's sexual availability will be assessed by women. There is more insight to be garnered about further specific sex acts.

### Effects of Individual Differences

During our analyses, we included several covariates that one might expect to influence how people use information about sexual history including religiosity, sexual disgust, and sociosexuality. Our third prediction regarding the effect of these individual differences was supported on an overall level. Higher levels of religiosity and sexual disgust, and more restricted sociosexuality were all associated with more negative appraisals of targets with a sexual history. Contrary to our expectation however, the effect of religiosity was not limited to short-term sexual relationships. Overall, while there was evidence that these individual differences affect how sexual history information is used more broadly, these did not seem to enhance or reduce SDS or sexual hypocrisy effects.

Overall, these findings, although original, dovetail neatly with the general finding in the literature that people rarely express the traditional double standard when they judge sexually active others. Further, considering both sexes in both MCs reveals predictable sex differences, where especially men are less negative toward sexually active women in a short-term context. Sexual availability is considered attractive and signaling this is an effective way for women to self-promote or flirt in short-term contexts (Bendixen & Kennair, 2015; Kennair et al., 2022).

The most interesting aspect of these findings may be that so many expect to find the traditional pattern expressed in modern society. An implicit negative attitude toward short-term sexual relations might be part of the explanation of why people continue to believe in the traditional sexual double standard. Intrasexual competition between women is probably also a driving mechanism, attempting to downregulate inflation for sexual access. However, the narrative might be leftover norm expectations from an era when there actually was more sexual control over women than men, for example because of religiosity. There are two aspects of the current findings that suggest that this explanation may be too simple. First, while the participants in the current study are from a highly secularized society, egalitarian and sexually liberal society compared to the United States (Bendixen et al., 2017), there are similar findings of reversed double standards or single standards in US samples, too (Crawford & Popp, 2003). Also, religiosity did generally influence the pattern of results for the SDS (although for own pursuit, religiosity was a robust covariate), although religious men were more critical of women for the socially undesirable behaviors factor. This question probably needs resolving with data from even less egalitarian, more religious, and less sexually societies. In the meantime, taking double sexual standards for granted and telling young women about the existence of such double standards, when indeed they might not exist, is probably more limiting for people's sexual liberty than other people's actual attitudes. Displaying a more sex-positive attitude, especially toward short-term sex, may be a better approach, than spreading the myths of traditional double standards and that primarily males are negative to an active female sexuality and agency.

### Limitations

The main limitation of the current work is that it was conducted on a convenience sample from a secular country which is high in sexual liberalism and has high gender egality. It is entirely possible that SDS are reduced in such countries and would reveal themselves more in countries which are more conservative and religious. Thus, a key future direction would be to replicate these findings in other countries to test for cross-cultural consistency, though often such research demonstrates that mating psychology is remarkably canalized (Thomas et al., 2020). Further, one variable that was not controlled for in the current study was degree of relatedness between friends and participants. Future studies might consider more social dimensions by including different degrees of genetic relatedness and social relations.

Despite sample characteristics, the random assignment procedure into short-term or long-term MCs and question-order manipulation ensures comparability of these factors. Another possible limitation is the comparison for testing hypocrisy; self-suitor versus same-sex friend appraisals that are not directly comparable regarding content. In the self-suitor appraisal, we asked the respondent to consider to what extent the target's sexual behavior reduced or increased the likelihood of pursuing ONS/relationship, while in the same-sex friend appraisal we asked the respondent to report the degree that their friend *should* pursue an ONS/relationship. The latter might appear more moralistic than the former.

## Conclusion

The current study considers both SDS and hypocrisy. We have different standards for our own versus same-sex friends’ partners, and this study suggests that people are more lenient toward friends’ partners. An active sexual history represents not only opportunities but is also a risk factor. It would seem we are more risk aversive for ourselves than for same-sex peers.

Women differentiate less between MCs, and a man's active sexual history thus reduces his partner value or attractiveness also in short-term contexts; this is the reversed double standard. For the long-term context there seems to be a single standard between the sexes, as both women and men assess men and women more negatively based on an active sexual history. However, for the short-term context, women are rated by both sexes as more attractive partners when they have an active sexual history. This suggests a context specific reversed double standard. This last finding is predicted from a sexual strategies perspective, and it highlights the need to consider the implicit values toward short-term mating in previous studies and highlights the importance of MC as specified by SST.

## Notes

1 The analyses were not affected by the exclusion of respondents aged above 30.
